# Assessment of the Impact of the Surface Modification Processes of Cotton and Polyester Fabrics with Various Techniques on Their Structural, Biophysical, Sensory, and Mechanical Properties

**DOI:** 10.3390/polym14040796

**Published:** 2022-02-18

**Authors:** Ewa Skrzetuska, Adam K. Puszkarz, Justyna Nosal

**Affiliations:** Institute of Material Science of Textiles and Polymer Composites, Faculty of Material Technologies and Textile Design, Lodz University of Technology, 116 Żeromskiego Street, 90-924 Lodz, Poland; ewa.skrzetuska@p.lodz.pl (E.S.); justynapytka@gmail.com (J.N.)

**Keywords:** biophysical comfort, sensory comfort, thermal insulation, flocking, screen printing, thermal-transfer printing, layer by layer, fabric, clothing, micro-CT

## Abstract

This article presents research on the assessment of the impact of surface modification of cotton and polyester fabrics using four techniques (flocking, layer by layer, screen printing and thermal-transfer printing) on their structural, mechanical, biophysical, and sensory properties. Depending on geometry and raw materials of the fabrics, the clothing made of them it is characterized by certain biophysical properties which are intended to protect the human body against external factors, but also against excessive sweating and overheating or cooling down. The aforementioned properties of the modified textiles were determined with: optical microscopy, microcomputed tomography, a tensile testing machine, sweating guarded-hotplate, air permeability tester, and the Kawabata evaluation system. Based on analysis of obtained results, it can be concluded that flocking reduces air permeability the most (−77% for cotton fabric and −99.7% for polyester fabric), and total hand value (−58% and −57%) and increases water vapor resistance the most (+769% and +612%) while the screen printing increases the thermal resistance the most (+119% and +156%) compared to unmodified textiles. It can be concluded that, when modifying textile substrates, the area of modification and their size on clothing products should be carefully selected so as not to adversely affect the feelings of potential wearers.

## 1. Introduction

*Utility comfort* of clothing can be defined as the state of physiological, sensory, and physical compatibility between a human and the environment in which he lives. According to the above definition, utility comfort can be divided into three components: (1) *biophysical comfort*, (2) *sensory comfort*, (3) *mental comfort* [1].

The *biophysical comfort* is one of the most important criteria for the evaluation of clothing products, contributing to the heat balance between the human body and the environment in which the user is staying. The heat balance results are mainly from the correct regulation of the temperature of the human body and the correct exchange of heat and moisture between the human skin and the environment. Such an exchange also takes place through the clothing. The basic properties of clothing, determining physiological comfort, include thermal insulation, moisture sorption, water vapor permeability and air permeability. Knitted fabrics, woven fabrics, and non-wovens, constituting the elements of clothing, are textile products made up of individual fibers. In knitted fabrics and woven fabrics, the fibers form a yarn—a continuous, cylindrical structure obtained because of twisting the fiber strands in the spinning process. In contrast, in non-woven fabrics the single fibers occur independently and form a chaotic layout. In knitted fabrics, woven fabrics and non-wovens, the empty spaces between fibers are filled with air. Due to the complicated geometry of the three aforementioned textiles, the physical phenomena occurring in them are determined by both the physical parameters of the raw material of the textiles and the physical parameters of air, in which the product is submerged [1].

The *sensory comfort* is related to the mechanical properties of the clothing and is described by a dimensionless numerical parameter called total hand value (THV). It is related to the stimulation of touch sensors located on the body because of contact between the human skin and the material from which the clothing is made [1].

The mental comfort is the result of physiological comfort and sensory comfort but is also related to the aesthetics and taste of the user. The lack of a sense of physiological and sensory comfort reduces the sense of mental comfort. However, mental comfort is a much wider concept. The psychological feelings of a human are also influenced by the stimuli he receives from the environment, not related to the surrounding conditions or clothing [1].

Research on the utility comfort of clothing focuses primarily on biophysical comfort and sensory comfort because, unlike mental comfort studies, they are based on an objective analysis of measurable clothing parameters determined in accordance with strict standards. The key parameters of clothing that determine the biophysical comfort, such as thermal insulation, thermal resistance, water vapor resistance, and water vapor permeability, are the subject of purely experimental studies [2,3,4,5,6,7,8,9,10] as well as experimental studies supported by theoretical research based on simulations using geometrical models of real textiles and of physical phenomena occurring inside them [11,12,13,14,15,16,17,18,19,20,21,22,23,24,25].

One of the factors influencing both the biophysical and sensory comfort of clothing is the surface modification. There are many reasons why the surfaces of clothing textiles are modified. One of the goals of the modification is to improve the properties of functional clothing parameters, affecting the safety of a selected group of users working in extremal environmental conditions like firefighters, rescuers, steel workers [26,27,28,29,30] as well as soldiers and policemen [31,32,33,34,35,36]. Surface modification is also commonly used in garments with healing properties [37,38,39,40,41,42] as well as smart clothing textiles that monitor the user’s life processes [43,44,45,46,47,48]. Surface modification is also commonly used for decorative clothing purposes [49,50,51,52,53].

The current article describes the research on the influence of four surface modification techniques of two clothing textiles (natural–cotton fabric and synthetic–polyester fabric) on their biophysical and sensory comfort. Such a selection of textiles for research was motivated by the fact that cotton and polyester are the most frequently used raw materials in clothing products. Cotton is the most commonly used natural raw material in the clothing industry due to its properties that determine its good air permeability and thermal insulation, while polyester is the most popular raw material used in the textile industry, among others. due to the fact that it can be obtained from recycling. Fabrics (and not knitted fabrics, for example) were selected because they are tensile-resistant and deformed during modification with selected printing techniques, which could result in the destruction of the applied layers and deformation of the pattern, etc.

The novelty of this work is the analysis of the influence of various modification techniques of textile materials on their final functional and sensory properties. In addition, the authors decided to conduct a wide range of studies to comprehensively verify their biophysical and sensory comfort after processes related to decorative and identification techniques in clothing.

These textiles were modified with the following methods: (1) flocking, (2) layer by layer, (3) screen printing, (4) thermal-transfer printing. The following parameters were determined for the unmodified and modified textiles: thermal resistance, water vapor resistance, air permeability (all describing biophysical comfort) and total hand value (sensory comfort). X-ray microtomography (micro-CT) and optical microscopy (OM) were used to investigate the relationship between the structure of modified textiles and their biophysical and sensory properties. The characteristics of the applied surface modification methods as well as measurement techniques are presented in Section 2.2.

## 2. Materials and Methods

### 2.1. Materials

**Figure 1:**
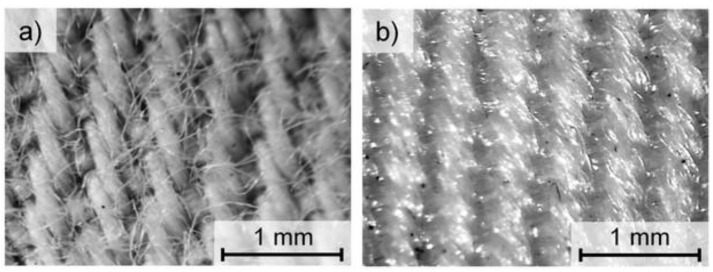
Optical microscopy images of cotton fabric (**a**) and polyester (PES) fabric (**b**).

Two fabrics made of cotton and polyester (PES) were the subject of the research. The characteristics of both fabrics made by Trans-Tex (Rzgów, Poland) are shown in Table 1, while Figure 1 shows the optical microscopy image of the fabrics. Both tested fabrics had the same weave and the same order of magnitude in thickness, surface mass and yarn porosity. The choice of cotton and polyester as fabric raw materials was aimed at verifying the modification of textiles made of several types of fibers (natural and synthetic).

### 2.2. Methods

#### 2.2.1. Modification Methods

##### Flocking

**Figure 2:**
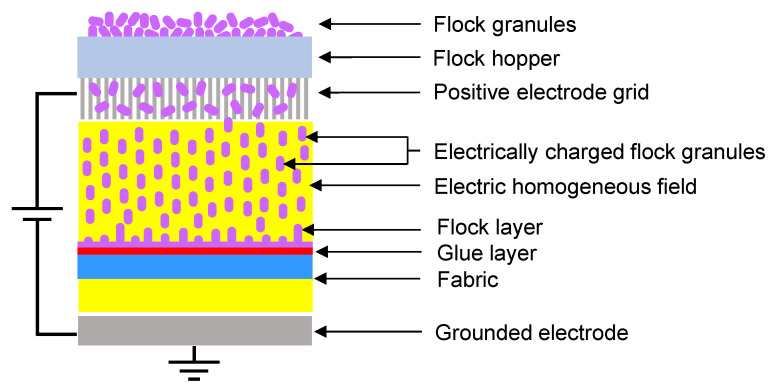
Scheme of applied flocking method.

The flocking method uses an electrostatic field to accelerate and spatially orientate electrically charged flock (short textile fibers or powder) deposited on the adhesive substrate (scheme of the method was presented in Figure 2). The flocking method can be used to modify the surfaces of textiles, wood, paper, foil, plastic, metal, plaster, glass, cardboard, concrete, rubber, and others. The method is widely used in the automotive industry (carpets, upholstery elements, storage compartments, cockpits, gaskets, soundproofing, etc.), textile (prints, patterns, etc.), toys (artificial grass, hair, etc.), jewelry industry (cassettes, boxes, packages), collectors (e.g., inside drawers for coins), furniture, as well as in the production of various types of packaging, boxes, bottles, stampings (as a decorative element and at the same time protecting the object). Deposited flock layer as a material is antistatic, abrasion-resistant, sound-absorbing, resistant to washing and cleaning, and has thermal insulation and filtration properties [56,57,58,59,60].

Before the flocking process, the surface of both fabrics was covered with a glue layer (BONAPUR^®^ FPU is a polyurethane adhesive made of special modified thermoplastic polyurethanes. Viscosity: about 150 mPas in accordance with PN-EN 12092, density: approximately 0.8 g·cm^−^^3^) using a screen-printing method. The fabrics were placed on a metal plate (grounded electrode). The surface of both tested fabrics was modified with the device Elektrostat FWN 04 (made by FLOK F.W., Lodz, Poland) using a NORIT^®^ SX2 (powder, from peat, multi-purpose activated charcoal, steam activated, and acid washed) in the form of granules (molar mass: 12.01 g·mol^−1^) as a flock.

##### Layer by Layer

The layer-by-layer technology is the most frequently used method of surface modification to give them the desired functional properties like structural, chemical [61], biological [62,63], optical [64], electrical [65], or magnetic [66]. The technology has many key advantages over other surface modification methods. One of the most important technologies is its simplicity of use (no complicated accessories are required) and low costs. In addition, it allows the use of a variety of deposited substances for multi-layer surface modification. In addition, the layer-by-layer technology is independent of the size and shape of the substrate, which means that it can be used not only on flat substrates, but also on substrates of various shapes, such as spheres and fibers [67].

**Figure 3:**
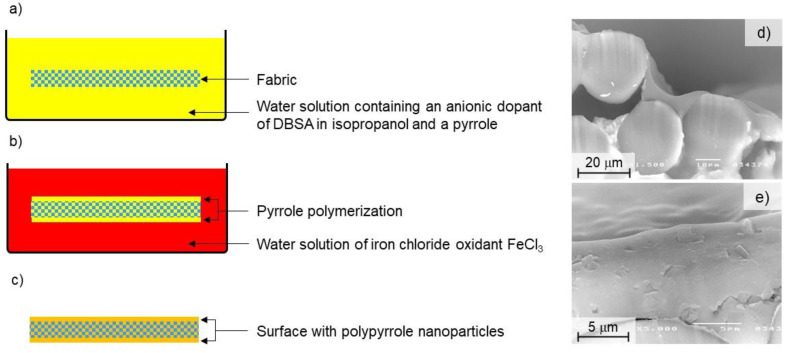
Scheme of applied layer by layer chemical method (**a**–**c**); scanning electron microscopy (SEM) images with polymerized pyrrole inside fibers (**d**) and on outer surface of fibers (**e**).

The applied layer-by-layer chemical method consisted of three stages. First, both fabrics were completely immersed in a water solution containing an anionic dopant of dodecylbenzene sulfonic acid (DBSA) in isopropanol at a concentration of 0.25 M and a pyrrole with a concentration of 1 M (Figure 3a). Then, the fabrics were immersed in a water solution of iron chloride oxidant (FeCl_3_) at a concentration of 0.25 M; as a result, pyrrole polymerization takes place on the fabric surface (Figure 3b). Then, after taking the fabrics out of the second solution, they were dried and a layer of polypyrrole nanoparticles remained on their surface (Figure 3c). In Figure 3d,e, scanning electron microscope (SEM) images with polymerized pyrrole on outer surface of fibers and inside fibers are presented.

##### Screen Printing

**Figure 4:**
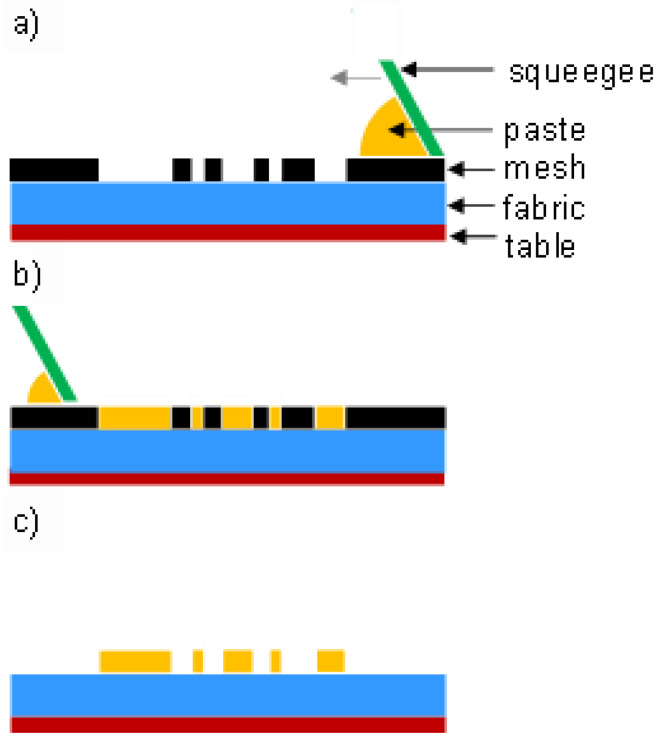
Scheme of screen printing. (**a**) applying paste on the mesh; (**b**) forcing the paste through the mesh; (**c**) screen printed fabric.

Screen printing is an exceptionally durable method of printing the print on the surface of various materials (textiles, foil, cardboard, metals, plastics, wood) by forcing the paste into the material, thus creating a uniform whole with the substrate. The printing form is a template (mask) placed on a fine mesh, woven, metal or made of synthetic fibers (mesh). The printout consists in forcing the paste through the mask and mesh with a squeegee (Figure 4). The advantage of this method is high color fastness and resistance to washing and abrasion. The technology is most often used to decorate light clothing such as T-shirts or pajamas [68,69,70], functional clothing [71], but it is also used to modify surfaces other than clothing textiles.

The MS-300 FRO (Printing Machine, Poznań, Poland) device was used to modify the surface of both fabrics, using carbon nanotube-based printing ink (AquaCyl AQ0301) and a polymer mesh (type: 60T made by SATTI) with an aluminum frame. A water dispersion was used made of carbon nanotubes with the trade name AQUACYL™ AQ0302 by Nanocyl (Sambreville, Belgium). This dispersion contained 3% of multi-wall carbon nanotubes (MWCNT) from the NC7000™ line, with average diameters of 9.5 nm, average lengths of 1.5 μm and a purity of 90%

##### Thermal-Transfer Printing

Thermal-transfer printing is a commonly used digital printing method in which ink is applied to a substrate by using a tape on which ink is previously applied. The printout is created when the heated tape with the ink applied is mechanically pressed against the substrate. Thermal transfer printing is widely used in many branches of science [72,73,74,75], industry and trade. Due to the durability of the print and the possibility of printing on various surfaces, it is used for labeling goods in supermarkets, marking clothing, printing markings in the chemical industry and healthcare, markings on products that must be clear and permanent.

**Figure 5:**
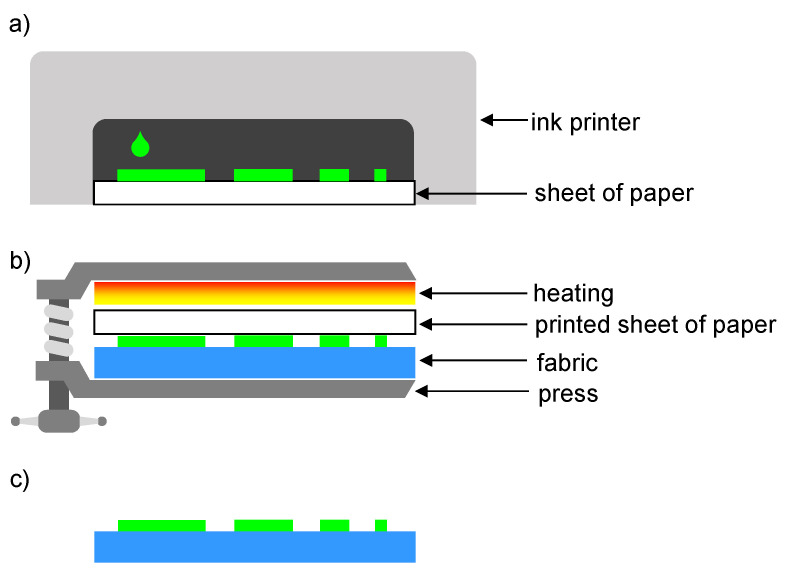
Scheme of thermal-transfer printing: (**a**) transferring the graphics to a paper substrate using the printer; (**b**) transferring graphic from paper to fabric using heat and press, (**c**) thermal transfer printed fabric.

A piezoelectric inkjet printer and a press BluePRESSLine were used to modify the fabrics. The first stage of the applied method of fabric surface modification was to transfer the prepared graphics to a paper substrate using the printer and sublimation ink PAPIJET LTI (Figure 5a). Thereafter, the printed paper was arranged over the fabrics so that the printed surface is in contact with the surface of the fabrics. The printed paper was placed together with the fabric in a heated press (Figure 5b) where the surface of the fabrics was modified due to the applied thermal and mechanical stress (Figure 5c).

#### 2.2.2. Evaluation of Modified Fabrics Properties

##### Structural Properties

X-ray Micro-Computed Tomography (Micro-CT)

Structural parameters (Table 1 and Table 2) and 3D reconstruction of the tested fabrics were determined using X-ray micro-computed tomography (SkyScan 1272; Bruker, Kontich, Belgium). Micro-CT outcomes were obtained applying the following scanning conditions: X-ray source voltage 50 kV, X-ray source current 200 µA, pixel size 5 µm. A 180° rotation was performed with a rotation step of 0.1° and no filter was selected.

##### Optical Microscopy (OM)

Optical microcopy images of the surface-tested fabrics were obtained using an optical microscope (PZO, Warsaw, Poland) equipped with a digital optical camera (DLT-Cam PRO, Delta Optical, Warsaw, Poland) and software (DLT CamViewer (Warsaw, Poland)).

##### Biophysical Properties

Thermal and Water Vapor Resistance (Sweating Guarded-Hotplate Test)

**Figure 6:**
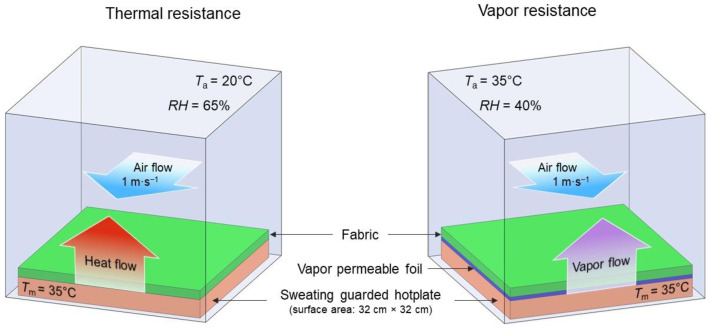
Conditions of textiles thermal and vapor resistance measurement.

The thermal and water vapor resistance of the tested fabrics were measured using a sweating guarded Hotplate 8.2 (made by Measurement Technology Northwest in Seattle, DC, USA) according to PN-EN ISO 11092:2014-11, ISO 7243. The parameter related to the evaluation of the thermal resistance *R_ct_* were computed according to the following equation:

(1) $$R_{ct}=\frac{\left(T_{m}-T_{a}\right)\cdot A}{H-\Delta H_{c}}-R_{ct0}$$

where: *T_m_*—heating plate temperature [°C]; *T_a_*—air temperature [°C]; *A*—surface of the measuring plate [m^2^], *H*—heating power supplied to the measuring plate [W], ∆*H_c_*—heating power correction in case of measuring thermal resistance [W], *R_ct_*_0_—instrument constant for measuring thermal resistance, [m^2^·°C·W^−1^] and the parameter related to the assessment of water vapor resistance *R_et_*_0_ [m^2^·Pa·W^−1^] is computed according to the equation:

(2) $$R_{et}=\frac{\left(p_{m}-p_{a}\right)\times A}{H-\Delta H_{e}}-R_{et0}$$

where: *p_m_*—saturated water vapor partial pressure [Pa] at the surface of the measuring plate at the temperature *T_m_*; *p_a_*—partial pressure [Pa] of water vapor in the air in the measuring chamber at the temperature *T_a_*; *A*—surface of the measuring plate [m^2^], *H*—heating power [W] supplied to the measuring plate; ∆*H_e_*—heating power correction [W] in the case of measuring the water vapor resistance, *R_et_*_0_—instrument constant [m^2^·Pa·W^−1^] for the measurement of water vapor resistance according to ISO 7243. The measurements were performed under the following constant conditions: thermal resistance: *T_a_* = 20 °C, *RH* = 65%, air flow speed 1 m·s^−1^; water vapor resistance: *T_a_* = 35 °C, *RH* = 40%, air flow speed 1 m·s^−1^ (Figure 6).

##### Air Permeability (Air Permeability Tester)

**Figure 7:**
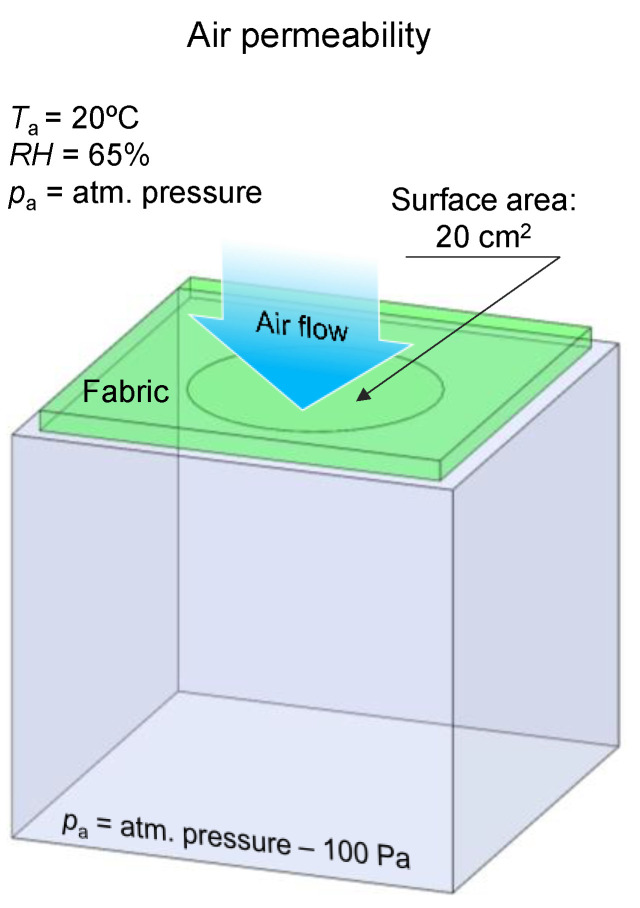
Conditions of air permeability measurement.

The measurements of air permeability of tested fabrics were performed using an air permeability tester (FX 3300 model made by Textest Instruments in Schwerzenbach, Switzerland) according to PN-EN ISO 9237:1998 in following constant conditions: relative humidity—*RH* = 65%, air temperature—*T_a_* = 20 °C, and air pressure—*p_a_* = 1013.25 hPa (Figure 7).

The tester maintains a constant pressure difference (100 Pa) between the reservoir and the environment, which causes a constant air flow through the tested fabric perpendicular to its surface. The device measures air flow leaving the textile. The air permeability of each fabric was evaluated as the average of 10 independent measurements. The surface area of the tested fabrics was 20 cm^2^.

##### Sensory Properties

Koshi, Numeri, Fukurami, Sofutosa, Total Hand Value (Kawabata Evaluation System—KES)

Sensory properties of tested fabrics were determined using the Kawabata evaluation system (KES) which is a set of devices applied to measure those textile material properties that enable predictions of the aesthetic features felt by a human touch. KES devices quantify tactile qualities of clothing textiles through features measurement of the mechanical properties related to comfort perception. Applying low forces, as in manipulating/touching clothing textiles, the KES determines the function played by bending rigidity (flexing), compression (thickness, softness), tensile (stretch), shear stiffness (drape), surface friction and roughness (next to skin) on tactile sensations. Based on a computational analysis of the measured mechanical properties mentioned above, KES provides an understanding of how the properties of the fibers, yarns, weave and the overall structure of the clothing textile and its finish affect the wearer’s sensory comfort.

To determine the field of application of the tested fabrics, the equation KN-201-MDY was chosen, referring to textiles used for women’s clothing of medium thickness (summer clothes). Using the above equation, apart from the evaluation of the total grip, the obtained results were related to the feeling of stiffness, smoothness, fullness and softness (Koshi, Numeri, Fukurami, Sofutosa).

Kawabata’s team assessed various THV characteristics for groups of materials used in selected clothing ranges, including summer and winter men’s clothes, thick and thin women’s coats. The basic features determining the suitability and quality of certain materials include: stiffness, smoothness and fullness [76].

Stiffness (from the Japanese: Koshi), a sensation related primarily to bending stiffness. This feeling is enhanced by the elastic properties of the material, i.e., all fabrics characterized by high density, or fabrics made of elastic and resilient yarns. The feeling of smoothness (from the Japanese: Numeri) is related to the sum of the characteristics of smoothness, softness and frizz. Materials made of cashmere wool have a high value for this feeling. The fullness (from the Japanese: Fukurami) is created by fullness and softness, it is closely related to the elasticity of materials subjected to compressive deformation, thickness or the feeling of a feeling of fullness THV.

This set of features is used in the study of groups of materials used in men’s autumn and winter clothing. Additionally, the sensations of Shari(crispness) and Hari (anti-drape stiffness) are considered for materials intended for summer men’s clothes.

When analyzing the assortment addressed to the opposite sex, two groups of materials with an intermediate thickness and thin materials were taken into account. It is the thickness that is the basic condition for belonging to a given group. There are four main features of the shank defined for medium thickness materials and six for thin materials.

In each of these cases, in addition to the previously defined features for fabrics intended for the men’s range, additional ones have been developed that play an important role in the selection of women’s clothes. Taking into account the groups of textile products intended for women’s garments of medium thickness, in addition to stiffness, smoothness and fullness, an additional feature called softness (from the Japanese: Sofutosa) was determined.

The feeling of softness is related to the interaction of three features: flexibility, plumpness and smoothness. Textiles intended for thin garments are characterized by stiffness, smoothness, fullness anti-drape stiffness, as well as scroop and flexibility. In order to correctly assess the quality of textile products according to the method proposed by the Hand Evaluation and Standardization Committee (HESC), the material must be correctly assigned to a given group. As a result of the research conducted, a library of patterns was developed for each of the basic features, depending on the intensity of feelings. According to such a scheme, the THV is assessed. The rank 5 was assigned to the materials with the perfect THV, while the samples that were not suitable for the particular type of garment were assigned the value of 0 [76].

##### Mechanical Properties

Breaking Force, Elongation at Brake (Tensile Testing Machine)

Mechanical properties of tested fabrics were determined using a tensile testing machine (Instron, model 4204 made in Norwood, MA, USA). Breaking force and elongation at break for unmodified and modified fabrics were determined according to PN-EN ISO 13934-1:2013. The size fabric samples were 250 mm × 50 mm. The tests were performed at a crosshead speed of 100 mm·min^−1^. The measurements of both mechanical parameters were taken in the direction of the warp and weft.

## 3. Results

This section presents the results and discussion of research on structural parameters and biophysical, sensory, mechanical properties of tested fabrics.

### 3.1. Structural Properties

**Figure 8:**
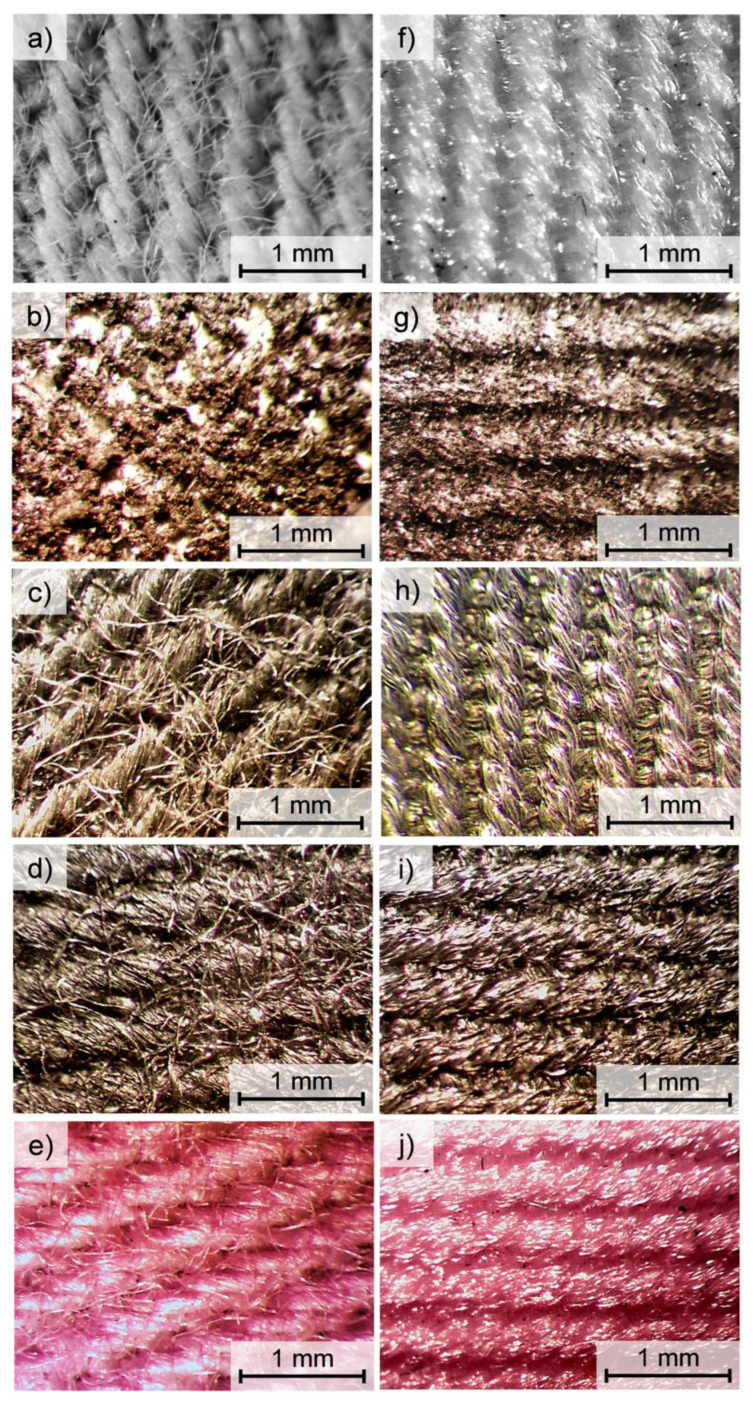
Optical microscopy images of cotton fabric ((**a**)—unmodified, (**b**)—flocked, (**c**)—layer by layer modified, (**d**)—screen printed, (**e**)—thermal transfer printed) and PES fabric ((**f**)—unmodified, (**g**)—flocked, (**h**)—layer by layer modified, (**i**)—screen printed, (**j**)—thermal transfer printed).

In Figure 8 optical microscope images of unmodified and modified surfaces of the tested fabrics using four selected techniques were presented. For cotton fabric (Figure 8a–e) and the polyester fabric (Figure 8f–j), the color of the dye used for each modification technique can be observed. In the case of fabrics modified with the flocking technique (cotton fabric—Figure 8b, and PES fabric—Figure 8g), the layer of glue and deposited granules obscure the yarns in the weft and warp and severely limit the identification of both fabric weaves.

**Figure 9:**
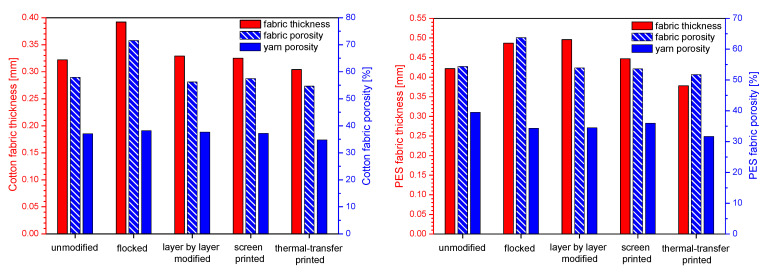
Structural parameters of unmodified and modified cotton fabric and PES fabric.

In Table 2 and Figure 9 structural parameters of unmodified and modified fabrics calculated using micro-CT are presented. The results obtained show that the applied four modifications affect the thickness of the modified fabric, the yarn porosity, and the fabric porosity.

For the modified cotton fabric, the following fabric thickness changes were observed compared to the unmodified one: flocked (+22%), layer-by-layer modified (+2%), screen printed (+1%), thermal-transfer printed (−6%). In the case of yarn porosity, the following differences were observed: flocked (+3%), layer by layer modified (+2%), screen printed (0%), thermal-transfer printed (−6%), while for the fabric porosity the following changes were observed: flocked (+23%), layer by layer modified (−3%), screen printed (−1%), thermal-transfer printed (−6%).

For the modified PES fabric, the following fabric thickness changes were observed compared to the unmodified one: flocked (+15%), layer-by-layer modified (+18%), screen-printed (+6%), thermal-transfer printed (−10%). In the case of yarn porosity, the following differences were observed: flocked (−13%), layer by layer modified (−13%), screen printed (−9%), thermal-transfer printed (−20%), while for the fabric porosity the following changes were observed: flocked (+17%), layer by layer modified (−1%), screen printed (−1%), thermal-transfer printed (−5%).

**Figure 10:**
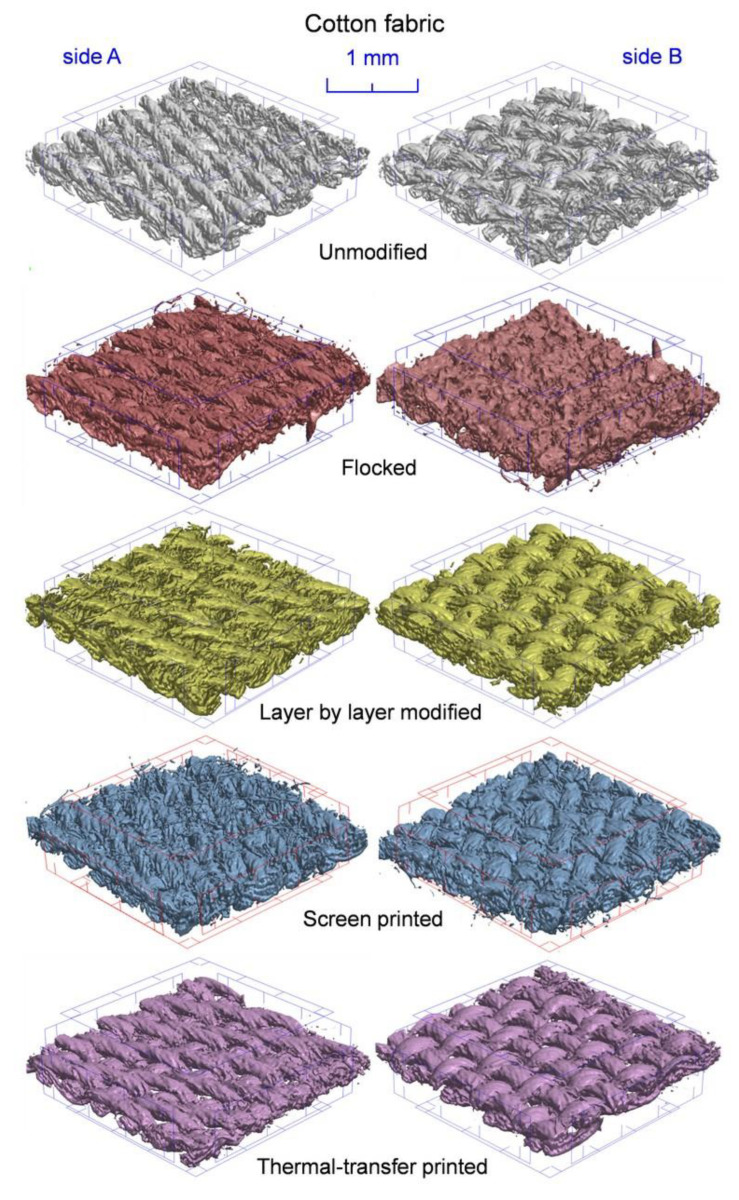
Three-dimensional micro-CT reconstruction of unmodified and modified cotton fabric.

**Figure 11:**
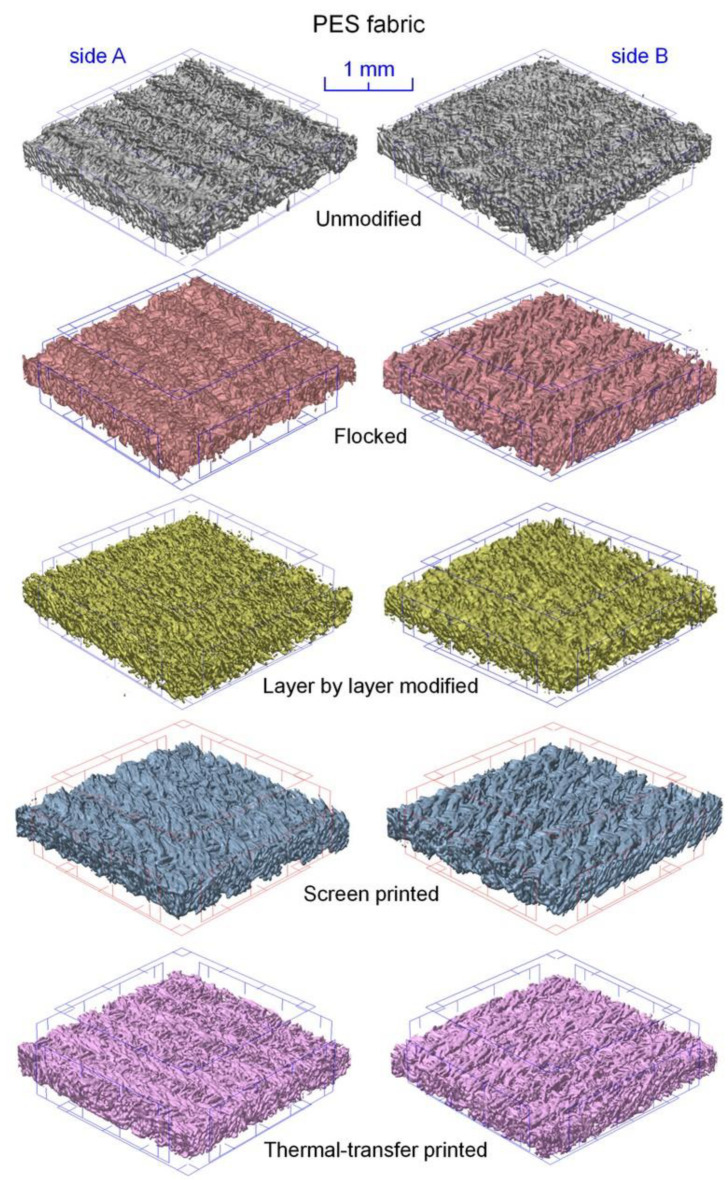
Three-dimensional micro-CT reconstruction of unmodified and modified PES fabric.

Computed microtomography 3D reconstruction of unmodified and modified textiles (cotton fabric—Figure 10, PES fabric—Figure 11) was obtained. In order to compare the effects of the applied modification methods, all the 3D reconstructions show fabrics with a surface area reduced to a square with sides 3 mm × 3 mm. The 3D reconstructions showed a modification effect on both sides of tested fabrics (side A—unmodified, side B—modified).

**Figure 12:**
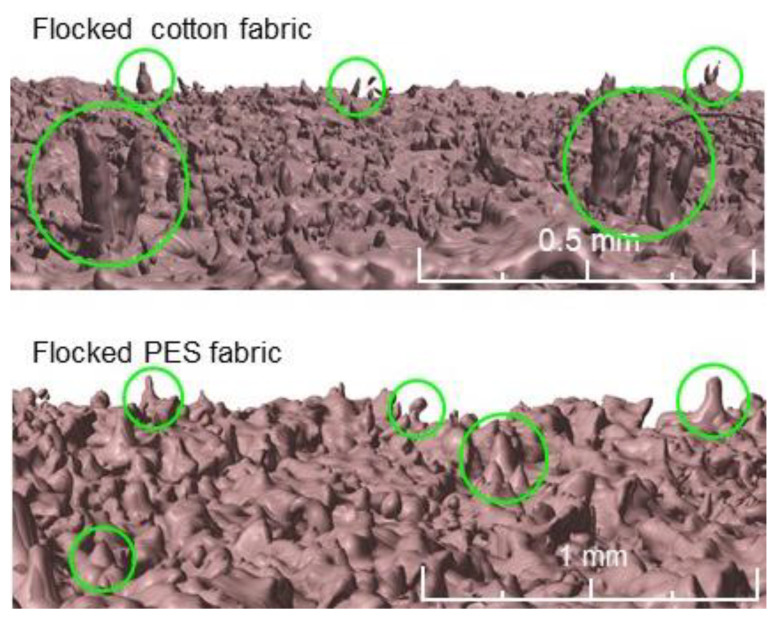
Three-dimensional micro-CT reconstruction of flocked surface of tested fabrics. Selected objects made of stuck granules formed a flocked fabric surface in the form of cones oriented vertically with the tips indicated inside the green circles.

In the case of both tested fabrics, the flocking modification one-sided effect is clearly visible. Side B, as a result of being covered with a continuous layer of glue, was almost completely non-porous, and as a result of applied electrodeposition, objects made of stuck granules were formed on its surface in the form of cones oriented vertically with the tip up. These cones were additionally reconstructed at a higher magnification in Figure 12. In the case of both tested fabrics, the layer-by-layer modification effect is double-sided, although clearly stronger on the B side. The reason for this may be that the modified fabric adhered on the A side to a smooth substrate, which hindered the expansion of nanoparticles on this side of the fabric. In the case of a both tested fabrics, the screen and thermal transfer printing effect is also one-sided. The modified B side is characterized by a significantly thicker yarn caused by the absorption of paste during screen printing and ink during thermal transfer printing. In addition, both fabrics modified with these two techniques are characterized by a less fluffy structure caused by the squeegee pressure during screen printing and the use of the press during thermal transfer printing.

### 3.2. Biophysical Properties

**Figure 13:**
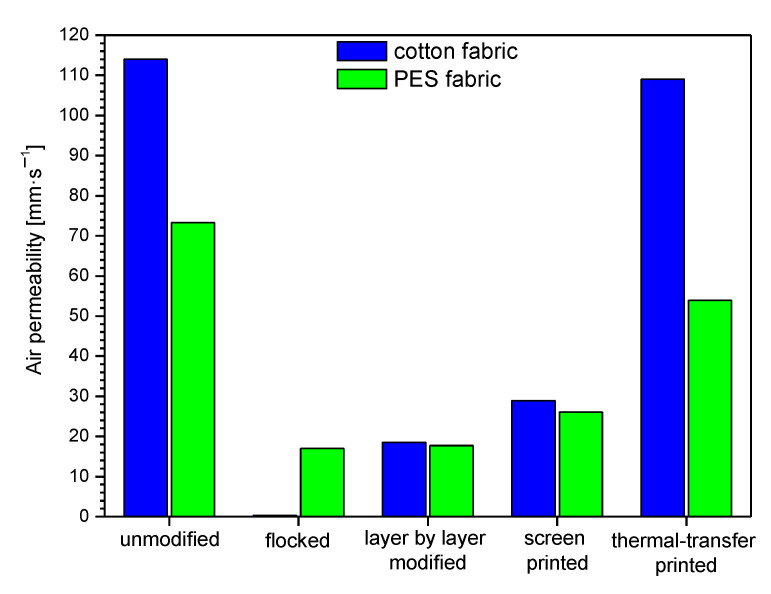
Air permeability of unmodified and modified fabrics.

**Figure 14:**
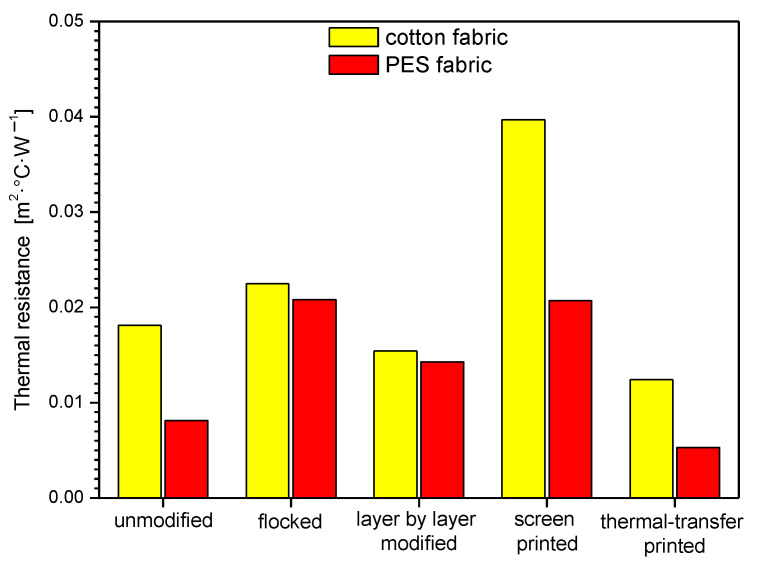
Thermal resistance of unmodified and modified fabrics.

**Figure 15:**
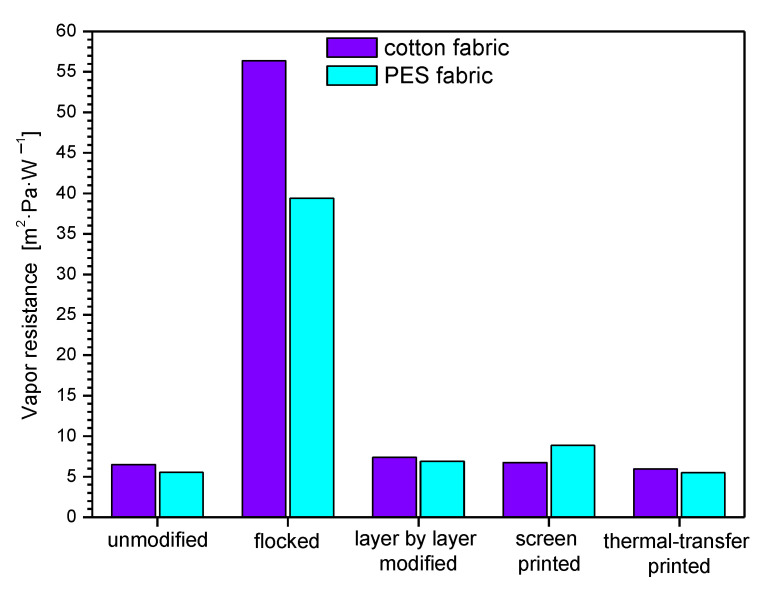
Vapor resistance of unmodified and modified fabrics.

In Table 3 and Figure 13, Figure 14 and Figure 15, biophysical parameters of unmodified and modified fabrics determined using an air permeability tester and sweating guarded-hotplate tester were presented. The results obtained show that the applied four modifications affect the air permeability, thermal resistance, and vapor resistance of the modified fabrics.

By analyzing the test results presented in Table 3 and Figure 13, it can be observed that the smallest differences in air permeability of modified fabrics compared to unmodified ones occur for thermal transfer-printed fabrics. In the case of the modified cotton fabric, the air permeability reached 109 mm·s^−1^, while for the unmodified one, 114 mm·s^−1^. In the case of the PES fabric modified with the same technique, the air permeability was 53.90 mm·s^−1^, while for the unmodified one, 73.30 mm·s^−1^.

The greatest differences in air permeability of modified fabrics compared to unmodified ones occur for flocked fabrics on the surface of which glue is evenly distributed over the surface, and then graphite granules are deposited. As a result of this modification of the surface of the textile product, the glue and granules fill the voids between the fibers, which reduces air permeability of flocked textile. As a result of modification with flocking, the greatest drops in air permeability were observed in the case of both tested fabrics: 0.35 mm·s^−1^ (cotton fabric) and 17.01 mm·s^−1^ (PES fabric). Fabrics modified with the layer by layer technique are also characterized by a significant decrease in air permeability, which can be observed in the case of polyester fabric, for which this parameter after modification is 17.70 mm·s^−1^, while for the modified cotton fabric it is 18.50 mm·s^−1^. In the case of layer-by-layer modification, during the polymerization process polypyrrole polymer structures are formed and the fibers of the fabric swell, which reduces the free air spaces between the fibers and reduces the fabric’s air permeability.

Summarizing the results obtained for the modified cotton fabric, the following air permeability percentage changes were observed compared to the unmodified one: flocked (−99.7%), layer-by-layer modified (−84%), screen printed (−75%), thermal-transfer printed (−4%). For the modified PES, fabric percentage changes were as follows: flocked (−77%), layer by layer modified (−76%), screen printed (−64%), thermal-transfer printed (−26%).

By analyzing the measurements results presented in Table 3 and Figure 14 it can be seen that screen-printed cotton fabric (0.0397 m^2^·°C·W^−1^) and flocked PES fabric (0.0208 m^2^·°C·W^−1^) have the highest thermal resistance among the modified fabrics, while layer-by-layer modified cotton fabric (0.0154 m^2^·°C·W^−1^) and thermal transfer printed PES fabric (0.0053 m^2^·°C·W^−1^) were characterized by the thermal resistance closest to that of unmodified textiles (cotton fabric: 0.0181 m^2^·°C·W^−1^, PES fabric: 0.0081 m^2^·°C·W^−1^).

Summarizing the obtained results for the modified cotton fabric, the following thermal resistance percentage changes were observed compared to the unmodified one: flocked (+24%), layer-by-layer modified (−15%), screen printed (+119%), thermal-transfer printed (−31%). For the modified PES fabric percentage changes were as follows: flocked (+157%), layer-by-layer modified (+77%), screen-printed (+156%), thermal transfer-printed (−35%).

By analyzing the measurements results presented in Table 3 and Figure 15 it can be seen that flocked cotton fabric (56.3704 m^2^·Pa·W^−1^) and flocked PES fabric (39.3716 m^2^·Pa·W^−1^) have the highest vapor resistance among the modified fabrics, while screen-printed cotton fabric (6.7534 m^2^·Pa·W^−1^) and thermal transfer-printed PES fabric (5.4799 m^2^·Pa·W^−1^) were characterized by the vapor resistance closest to that of unmodified textiles (cotton fabric: 6.4853 m^2^·Pa·W^−1^, PES fabric: 5.5276 m^2^·Pa·W^−1^).

The high resistance of flocked fabrics is because the pores are clogged during the glue application process, as a result of which the fabric neither absorbs water nor transmits water vapor. Fabrics modified using the other three techniques have values like those of unmodified fabrics.

Summarizing the obtained results for the modified cotton fabric, the following vapor resistance percentage changes were observed compared to the unmodified one: flocked (+769%), layer-by-layer modified (+14%), screen-printed (+4%), thermal transfer-printed (−8%). For the modified PES fabric percentage changes were as follows: flocked (+612%), layer-by-layer modified (+25%), screen-printed (+61%), thermal transfer-printed (−1%).

### 3.3. Mechanical Properties

**Figure 16:**
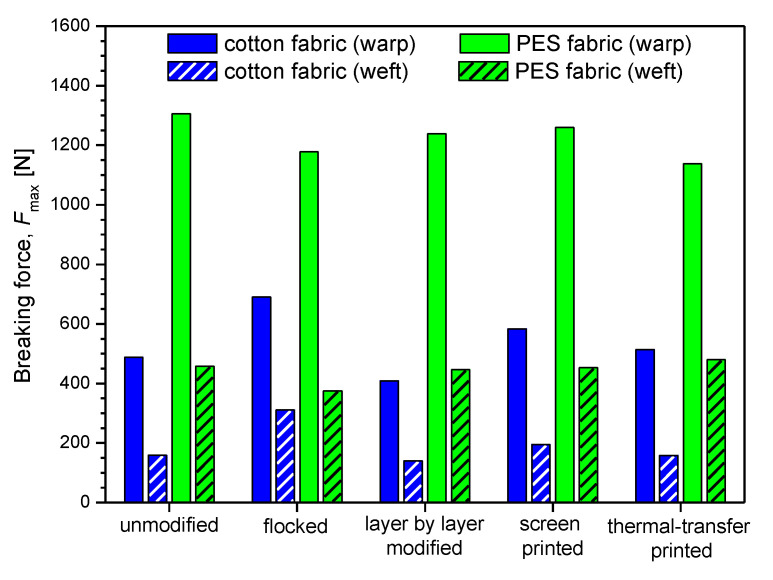
Maximum breaking force of unmodified and modified fabrics.

**Figure 17:**
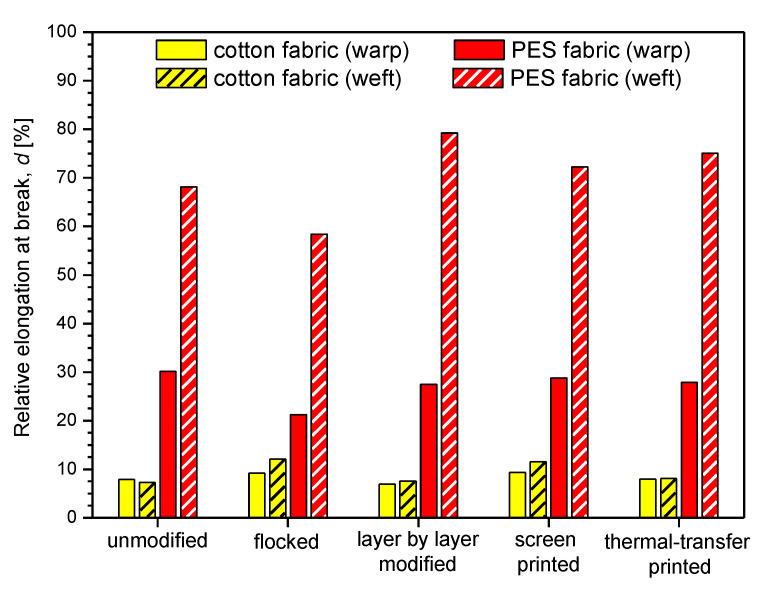
Relative elongation of unmodified and modified fabrics.

In Table 4 and Figure 16 and Figure 17 the mechanical parameters of unmodified and modified fabrics determined using tensile testing machine are presented. The obtained results show that the applied four modifications affect the breaking force and relative elongation at break of the modified fabrics.

By analyzing the measurements results presented in Table 5 and Figure 16 it can be observed that flocked cotton fabric (warp: 690.21 N, weft: 310.99 N) and screen-printed PES fabric (warp: 1259.25 N, weft: 446.90 N) have the highest breaking force among the modified fabrics, while thermal transfer-printed cotton fabric (warp: 513.62 N, weft: 157.82 N) and screen-printed PES fabric (warp: 1259.25 N, weft: 452.41 N) were characterized by the breaking force closest to that of unmodified textiles (cotton fabric—warp: 488.01 N, weft: 159.00 N, PES fabric—warp: 1305.66 N, weft: 458.06 N).

Summarizing the results obtained for the modified cotton fabric, the following breaking force percentage changes were observed compared to the unmodified one: flocked (warp: +41%, weft: +96%), layer-by-layer modified (warp: −16%, weft: −12%), screen-printed (warp: +20%, weft: +23%), thermal transfer-printed (warp: +4%, weft: −1%). For the modified PES fabric, percentage changes were as follows: flocked (warp: −10%, weft: −18%), layer-by-layer modified (warp: −5%, weft: −2%), screen-printed (warp: −4%, weft: −1%), thermal transfer-printed (warp: −13%, weft: +5%).

By analyzing the measurements results presented in Table 4 and Figure 17 it can be observed that flocked cotton fabric (warp: 9.21%, weft: 12.12%) and layer-by-layer modified PES fabric (warp: 27.47%, weft: 79.23%) have the highest breaking force among the modified fabrics, while thermal transfer-printed cotton fabric (warp: 7.98%, weft: 8.07%) and screen-printed PES fabric (warp: 28.78%, weft: 72.25%) were characterized by the breaking force closest to that of unmodified textiles (cotton fabric—warp: 7.91%, weft: 7.32%, PES fabric—warp: 30.17%, weft: 68.17%).

Summarizing the results obtained for the modified cotton fabric, the following relative elongation at break percentage changes were observed compared to the unmodified one: flocked (warp: +16%, weft: +66%), layer-by-layer modified (warp: −12%, weft: +3%), screen-printed (warp: +18%, weft: +58%), thermal transfer-printed (warp: +1%, weft: +10%). For the modified PES fabric percentage changes were as follows: flocked (warp: −30%, weft: −14%), layer-by-layer modified (warp: −9%, weft: +16%), screen-printed (warp: −5%, weft: +6%), thermal transfer-printed (warp: −8%, weft: +10%).

### 3.4. Sensory Properties

**Figure 18:**
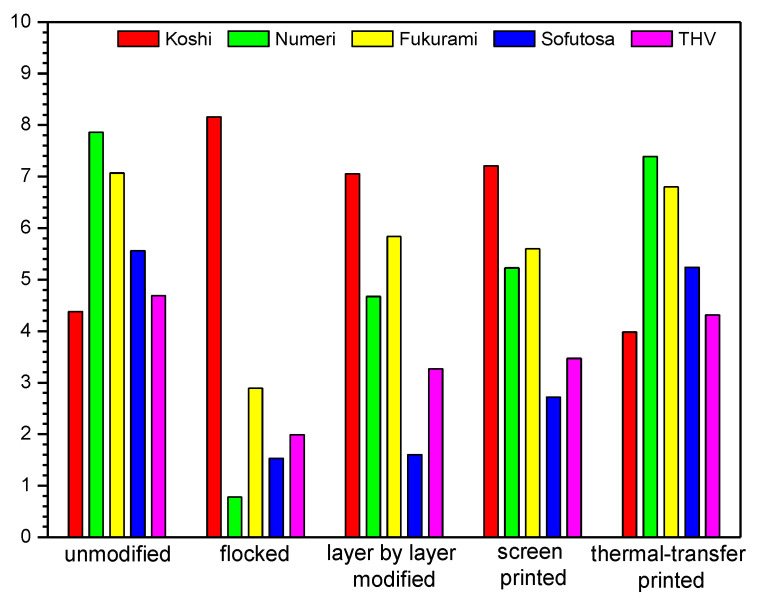
Sensory properties of unmodified and modified cotton fabric.

**Figure 19:**
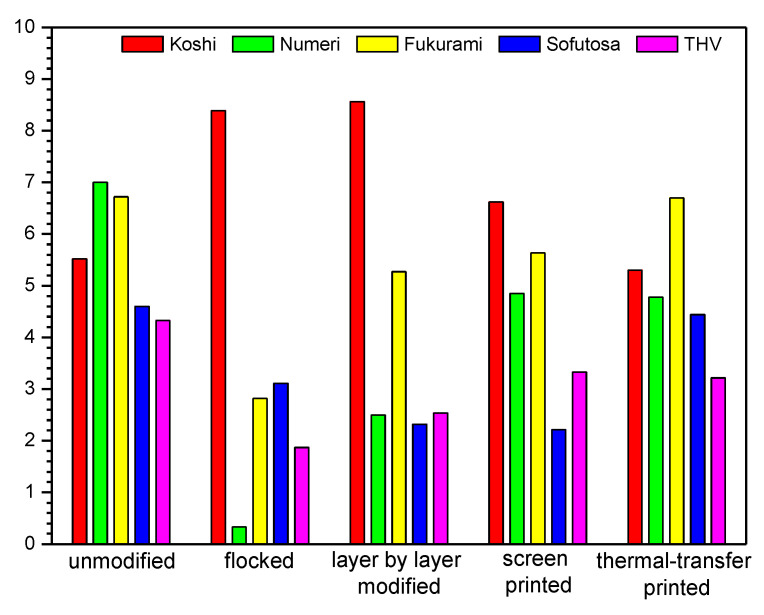
Sensory properties of unmodified and modified PES fabric.

In Table 5 and Figure 18 and Figure 19 sensory parameters of unmodified and modified fabrics determined using Kawabata evaluation are shown. The results obtained show that the applied four modifications affect all five analyzed sensory parameters (Koshi, Numeri, Fukurami, Sofutosa, Total Hand Value) of the modified fabrics.

Based on the results presented in Table 5, it can be concluded that the flocking modification, in which an adhesive was used to fix the granules, made the modified fabric very stiff. The smooth feel, which is related to the sum of the smoothness, softness and curl characteristics, is significantly worsened for flock-modified fabrics. The fullness, which is created by the bulk, is closely related to the elasticity of the materials subjected to compressive deformation, the thickness or the feeling of a warm grip, and it is also significantly deteriorated in the case of modification with the flocking technique. The fullness of softness is related to the interaction of three features: flexibility, plumpness, and smoothness, and similarly to the other parameters it deteriorates as a result of the flocking method.

Also, the screen-printing method, in which the printing paste is applied to the surface of the fabrics, increases the stiffness of the tested material and worsens the feeling of smoothness. The layer-by-layer technique, in which the material is immersed in solutions (as a result of which nanoparticles are built on the surface and between woven structures), causes an increase in bending stiffness, which affects the feeling of smoothness and softness.

Analyzing the data presented in Table 5 and Figure 18 and Figure 19, flocked textiles (cotton fabric: 8.16, polyester fabric: 8.39) stand out among modified fabrics, the highest stiffness (Koshi) is found in flocked fabrics (cotton fabric: 8.16, PES fabric: 8.39). Thermal transfer-printed fabrics achieved the lowest values, the closest to that of unmodified fabrics (cotton fabric: 3.98, PES fabric: 5.30). Among the modified fabrics, thermal transfer-printed cotton fabric (7.39) and screen-printed PES fabric (4.85) are the most smooth (Numeri), while flocked fabrics achieved the lowest smoothness (cotton fabric: 0.78, polyester fabric: 0.33). Among the modified fabrics, thermal transfer-printed textiles (cotton fabric: 6.80, PES fabric: 6.70) characterized by the highest fullness (Fukurami), while flocked fabrics achieved the lowest values (cotton fabric: 2.89, polyester fabric: 2.82). Out of the modified fabrics, thermal transfer-printed textiles (cotton fabric: 5.24, PES fabric: 4.44) characterized by the highest smoothness (Sofutosa), while flocked cotton fabric (1.53) and screen-printed PES fabric (2.21) obtained the lowest values. Among the modified fabrics, thermal transfer-printed cotton fabric (4.31) and screen-printed PES fabric (3.33) were characterized by the highest THV, while flocked textiles (cotton fabric: 1.99, PES fabric: 1.87) reached the lowest values.

Summarizing the results obtained for the modified fabrics, the percentage changes of sensory properties were calculated compared to the unmodified one. For the modified cotton fabric in the case of Koshi the following differences were found: flocked (+86%), layer-by-layer modified (+61%), screen-printed (+65%), thermal transfer-printed (−9%). In the case of Numeri, the following differences were observed: flocked (−90%), layer-by-layer modified (−41%), screen-printed (−33%), thermal transfer-printed (−6%), For Fukurami, the following differences were found: flocked (−59%), layer-by-layer modified (−17%), screen-printed (−21%), thermal transfer-printed (−4%). In the case of Sofutosa, the following differences were noticed: flocked (−73%), layer-by-layer modified (−71%), screen-printed (−51%), thermal transfer-printed (−6%), while for the THV the following changes were obtained: flocked (−58%), layer-by-layer modified (−30%), screen-printed (−26%), thermal transfer-printed (−8%). For the modified PES fabric, the following Koshi changes were observed: flocked (+52%), layer-by-layer modified (+55%), screen-printed (+20%), thermal transfer-printed (−4%). In the case of Numeri, the following differences were observed: flocked (−95%), layer-by-layer modified (−64%), screen-printed (−31%), thermal transfer-printed (−32%), For Fukurami, the following differences were found: flocked (−58%), layer-by-layer modified (−22%), screen-printed (−16%), thermal transfer-printed (−0.3%). In the case of Sofutosa, the following differences were noticed: flocked (−32%), layer-by-layer modified (−50%), screen-printed (−52%), thermal transfer-printed (−3%), while for the THV the following changes were obtained: flocked (−57%), layer-by-layer modified (−41%), screen-printed (−23%), thermal transfer-printed (−26%).

## 4. Discussion

**Figure 20:**
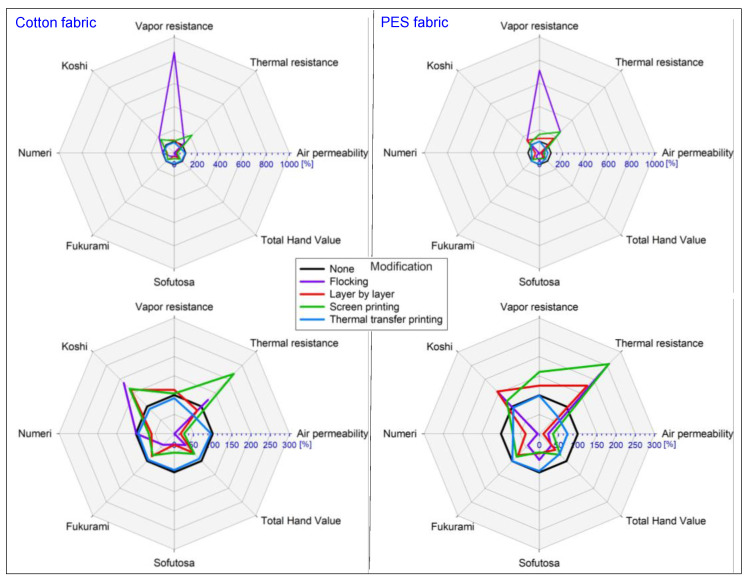
Relative percentage changes in the biophysical and sensory parameters of cotton and polyester fabric caused by the four applied modifications. Unmodified fabrics were treated as a reference (black plot—regular octagon).

Techniques for modifying the surface of textile substrates are an excellent basis for the development of decorative patterns as well as for marking finished products, e.g., with a company logo. However, it should be remembered that each modification introduced may affect the functional properties of the final textile product, its comfort of use and the feeling of touch to our skin (total hand value). Figure 20 presents the results of research on sensory and biophysical properties in order to better understand the problem related to the modification of the surface of textile products.

The results obtained for the air permeability show that that none of the applied four modifications increased the parameter of the fabrics in relation to unmodified fabrics; however, the greatest decrease in thermal resistance was observed for thermal transfer printed PES fabric (from 114.00 mm·s^−1^ to 0.35 mm·s^−1^; decrease: −99.7%). The results obtained for the thermal resistance show that the greatest rise in relation to unmodified fabrics was observed for the flocked PES fabric (from 0.0081 m^2^·°C·W^−1^ to 0.0208 m^2^·°C·W^−1^; rise: +156%). However, the greatest decrease in thermal resistance was observed for thermal transfer-printed PES fabric (from 0.0081 m^2^·°C·W^−1^ to 0.0053 m^2^·°C·W^−1^; decrease: −35%). The results obtained for the vapor resistance show that the greatest rise in relation to unmodified fabrics was observed for the flocked cotton fabric (from 6.4853 m^2^·Pa·W^−1^ to 56.3704 m^2^·Pa·W^−1^; rise: +769%). However, the greatest decrease in thermal resistance was observed for thermal transfer-printed cotton fabric (from 6.4853 m^2^·Pa·W^−1^ to 5.9743 m^2^·Pa·W^−1^; decrease: −8%). The results obtained for the Koshi show that the greatest rise in relation to unmodified fabrics was observed for the flocked cotton fabric (from 4.38 to 8.16; rise: +86%). However, the greatest decrease in Koshi was observed for thermal transfer-printed cotton fabric (from 4.38 to 3.98 m^2^·Pa·W^−1^; decrease: −9%). The results obtained for the Numeri show that that none of the applied four modifications increased the parameter of the fabrics in relation to unmodified fabrics however, the greatest decrease in Numeri was observed for flocked PES fabric (from 7.00 to 0.33; decrease: −95%). The results obtained for the Fukurami show that that none of the applied four modifications increased the parameter of the fabrics in relation to unmodified fabrics; however, the greatest decrease in Fukurami was observed for flocked cotton fabric (from 7.07 to 2.89; decrease: −59%). The results obtained for the Sofutosa show that that none of the applied four modifications increased the parameter of the fabrics in relation to unmodified fabrics; however, the greatest decrease in Sofutosa was observed for flocked cotton fabric (from 5.56 to 1.53; decrease: −73%). The results obtained for the total hand value show that that none of the applied four modifications increased the parameter of the fabrics in relation to unmodified fabrics however, the greatest decrease in THV was observed for flocked cotton fabric (from 4.69 to 1.99; decrease: −58%).

Based on the organoleptic evaluation of the tested fabrics, it can also be seen that these fabrics have become stiffer and coarser. Surface modification processes had a negative effect on air permeability. The largest decrease was recorded for flocked textiles, where the values for cotton fabric were close to 0 mm·s^−1^. This method has a significant impact on the structure of fabrics. The glue particles clogged the voids between the fibers of the fabric and the pores, making the textiles practically impermeable. A similar situation occurs in the case of film printing. Lower values of air permeability were also noted in the case of the layer-by-layer method, due to the swelling of the fibers.

Analyzing the data presented in Figure 20, it can be observed that thermal transfer-printed fabrics have the smallest changes in biophysical and sensory properties in relation to unmodified fabrics. In the case of thermal transfer-printed cotton fabric, only the thermal resistance was significantly reduced. Analyzing the results regarding thermal transfer-printed PES fabric, these show that air permeability, thermal resistance and total hand value have decreased.

The research results indicate a significant reduction in air permeability and an increase in water vapor resistance for fabrics modified with the layer-by-layer technique. This is because the modification with this method causes the fibers to swell, reducing the air spaces in the material structure. It also led to a deterioration of the sensory properties responsible for the total hand value. A similar situation occurs with the method of modification with the screen-printing technique, where in this case, under the influence of pressure and the use of printing paste, some of the pores of the fabrics were clogged, which reduced air permeability and increased thermal resistance and water vapor resistance, as well as material stiffness.

The most significant differences can be observed for the flocking technique, where air permeability was significantly reduced for both tested fabrics, and thus the water vapor resistance and stiffness increased significantly. This is because in the flocking process, the glue is first applied to the fabric surfaces, and then polymer granules or fibers are applied, which clog the gaps and pores of the fabrics.

It is not possible to use the same ink/paste/granules for different techniques used in the work. In the future, it is possible to try to use one basic carrier, e.g., carbon nanotubes. However, it should be remembered that each technique requires a different structure/consistency (the paste is thick and sticky, the ink has a low surface tension etc.) Applied thermal transfer printing requires a different chemical structure, so that the ink/paste/granulate is transferred from the paper to the material under the influence of temperature). Therefore, even the use of the same carrier will not allow clear determination of how a given carrier affects the properties of the clothing tested in the work.

The analysis of the authors’ earlier works showed that the changes in sensory comfort associated with the use of various printing pastes had a small impact on the result. In the work [46], two printing compositions were applied using the screen-printing technique. The first was an aqueous dispersion of carbon nanotubes under the trade name AQUACYL™ AQ0302 from Nanocyl, and the second printing composition was a mixture of AQUACYL™ AQ0302 mixed with Sigma Aldrich^®^ polypyrrole powder (CAS No. 30604-81-0). This publication showed that the total grip for a knitted fabric of 86% cotton/14% lycra printed with printing composition No. 1 was 3.80 while the second composition was 3.60. The authors conducted similar studies for ink-jet printing, where poly (3,4-ethylenedioxythiophene): poly (styrene sulfonate) (PEDOT: PSS) and NANO INK AX JP-60n (electrically conductive composition with silver fillers) were used. The analysis of the results shows that the applied ink did not cause any changes in the total nip (THV), as the cotton fabric samples before printing had a THV level of 3.31, and after printing the THV level for both inks was 3.69. In the case of thermal resistance tests for ink-jet printing, the results for the unprinted fabric were m^2^·°C·W^−1^, and after printing—0.0158 m^2^·°C·W^−1^ for the first ink, and 0.0159 m^2^·°C·W^−1^ for the second ink. Based on the above works, it can be concluded that changes in the composition of printing compositions, while using the same printing technique, do not significantly affect the biophysical and sensory comfort of the modified textile substrates.

## 5. Conclusions

Clothing plays an important role in the life of every person. Its main task is to protect against external factors: thermal (e.g., protection against overheating and cooling), chemical (e.g., protection of the skin against toxic substances), and mechanical (e.g., protection against skin cuts). Thanks to clothing, we can avoid many injuries, but also create our own image (aesthetic role of clothing). Many studies show that the constant and long-term contact of clothing products with the human body is not indifferent to its health. The performance of all these roles by clothing is referred to as the state of comfort.

Techniques for modifying textile surfaces no longer have a purely decorative role, but have become an information carrier. Thanks to the durability of the applications made by printing technology, they are very competitive in the textile industry. Manufacturers who want to be associated with reliability and high quality of the product use printing techniques to mark their products. Designers who are just beginning to find their way into the fashion industry often mark their products with print in order to stand out from other brands. However, it should be remembered to carefully select the area of modification and their size on clothing products, so as not to adversely affect the feelings of potential recipients.

The research results presented in this article have shown that each of the surface modification methods used affects the biophysical and sensory comfort of the user of the final product. The best method of modification seems to be the thermal-transfer printing method, as it is the least affected by the limitation related to the amount of coverage of the textile material. This method does not significantly change the breathability (−4% for cotton fabric and −26% for PES fabric) of the textile, total hand value (−8% for cotton fabric and −26% for polyester fabric), water vapor resistance (−8% for cotton fabric and −1% for polyester fabric) and thermal resistance (−31% for cotton fabric and −35% for polyester fabric) compared to unmodified textiles. The application of printing pastes to textile surfaces causes a reduction in air permeability, which is best illustrated by the example of a cotton fabric, which was 114 mm·s^−1^ before printing and 28.90 mm·s^−1^ after printing. The thermal resistance increased analogously from 0.0181 m^2^·°C·W^−1^ to 0.0397 m^2^·°C·W^−1^. Even more drastic changes were characterized by the flocking technique, where the air permeability for the cotton fabric decreased to the level of 0.35 mm·s^−1^, and the thermal resistance increased to 0.0225 m^2^·°C·W^−1^. In the case of polyester fabric, the changes in the unmodified sample were air permeability of 73.30 mm·s^−1^, respectively, and 17.01 mm·s^−1^ after flocking, and 26.10 mm·s^−1^ after film printing. As for the thermal resistance for unmodified fabrics, it was 0.0081 m^2^·°C·W^−1^, for flocked samples 0.0208 m^2^·°C·W^−1^, layer by layer 0.0143 m^2^·°C·W^−1^ and screen printing 0.0207 m^2^·°C·W^−1^. The smallest changes were observed for the thermal transfer printing technique, where air permeability decreased by about 20 mm·s^−1^ for the PES fabric, and the heat resistance by about 0.003 m^2^·°C·W^−1^. Water vapor resistance behaves inversely with air permeability. If the air permeability increases, the water vapor resistance decreases, which is shown by the result for cotton fabric, for which it was 6.4853 m^2^·Pa·W^−1^ before modification, and 56.3704 m^2^·Pa·W^−1^ after flocking.

In addition, it is a method that allows you to design a pattern using computer software and then easily transfer it to textile surfaces. In the case of screen printing and flocking techniques, it is necessary to make screens with which to create a pattern. This technique is economical only in the case of large-scale production, as each change of the pattern necessitates the preparation of new screens. The layer by layer method does not allow for the creation of patterns, but only complete changes to the modified substrate.

## Figures and Tables

**Table 1:** Characteristics of tested fabrics.

Nr	Textile Type	Weave	Composition	Thickness ^(a)^ [mm]	Surface Mass ^(b)^ [g·m^−^^2^]	Total Porosity ^(c)^ [%]	YarnPorosity ^(c)^ [%]
**1**	fabric	twill	cotton	0.32	143.74	57.94	37.07
**2**	fabric	twill	PES	0.42	157.92	71.53	39.55

(a) determined according to PN-EN ISO 5084:1999 [54]. (b) determined according to PN EN 12127:2000 [55]. (c) determined according to X-ray micro-CT (computed tomography).

**Table 2:** Structural parameters of unmodified and modified fabrics.

Textile	Modification Method	Micro-Computed Tomography		
		Fabric Thickness [mm]	Yarn Porosity [%]	Fabric Porosity [%]
Cotton fabric	None	0.322	37.069	57.937
	Flocking	0.392	38.195	71.532
	Layer by layer	0.329	37.652	56.197
	Screen printing	0.325	37.157	57.477
	Thermal-transfer printing	0.304	34.763	54.698
PES fabric	None	0.422	39.545	54.412
	Flocking	0.487	34.326	63.793
	Layer by layer	0.496	34.498	53.953
	Screen printing	0.447	35.951	53.635
	Thermal-transfer printing	0.378	31.655	51.739

**Table 3:** Biophysical properties of unmodified and modified fabrics.

Textile	Modification Method	Air Permeability Tester	Sweating Guarded-Hotplate Tester	
		Air Permeability, *P_AIR_* [mm·s^−1^]	Thermal Resistance, *R_ct_* [m^2^·°C·W^−^^1^]	Vapor Resistance, *R_et_* [m^2^·Pa·W^−1^]
Cotton fabric	None	114.00	0.0181	6.4853
	Flocking	0.35	0.0225	56.3704
	Layer by layer	18.50	0.0154	7.3775
	Screen printing	28.90	0.0397	6.7534
	Thermal-transfer printing	109.00	0.0124	5.9743
PES fabric	None	73.30	0.0081	5.5276
	Flocking	17.01	0.0208	39.3716
	Layer by layer	17.70	0.0143	6.9075
	Screen printing	26.10	0.0207	8.8755
	Thermal-transfer printing	53.90	0.0053	5.4799

**Table 4:** Mechanical properties of unmodified and modified fabrics.

Textile	Modification Method	Breaking Force, *F*_max_ [N]		Relative Elongation at Break, *d* [%]	
		Warp	Weft	Warp	Weft
Cotton fabric	None	488.01	159.00	7.91	7.32
	Flocking	690.21	310.99	9.21	12.12
	Layer by layer	408.37	140.46	6.95	7.56
	Screen printing	583.26	195.23	9.36	11.55
	Thermal-transfer printing	513.62	157.82	7.98	8.07
PES fabric	None	1305.66	458.06	30.17	68.17
	Flocking	1178.06	375.26	21.25	58.40
	Layer by layer	1239.26	446.90	27.47	79.23
	Screen printing	1259.25	452.41	28.78	72.25
	Thermal-transfer printing	1137.89	479.94	27.90	75.08

**Table 5:** Sensory properties of unmodified and modified fabrics.

Textile	Modification Method	Kawabata Evaluation System				
		Koshi	Numeri	Fukurami	Sofutosa	THV
Cotton fabric	None	4.38	7.86	7.07	5.56	4.69
	Flocking	8.16	0.78	2.89	1.53	1.99
	Layer by layer	7.05	4.67	5.84	1.60	3.27
	Screen printing	7.21	5.23	5.60	2.72	3.47
	Thermal-transfer printing	3.98	7.39	6.80	5.24	4.31
PES fabric	None	5.52	7.00	6.72	4.60	4.33
	Flocking	8.39	0.33	2.82	3.11	1.87
	Layer by layer	8.56	2.50	5.27	2.32	2.54
	Screen printing	6.62	4.85	5.64	2.21	3.33
	Thermal-transfer printing	5.30	4.78	6.70	4.44	3.22

## Data Availability

Not applicable.
